# Supplementary material for: Machine Learning in Antibody Diagnostics for Inflammatory Bowel Disease Subtype Classification
Source: Diagnostics (Basel). 2023 Jul 26;13(15):2491. doi: 10.3390/diagnostics13152491 (PMC10417520; doi:10.3390/diagnostics13152491)
Supplement: Supplementary file 1 [file diagnostics-13-02491-s001.zip › diagnostics-2486448-supplementary.pdf]

Supplementary Table S1. Clinical characteristics of adult CD and UC patients

	**CD**		**UC**	
number of patients	50		50	
males, n (%)	26 (52)		29 (58)	
age at diagnosis, median (IQR), y	24 (19-34.5)		34.5 (27.8-48.8)	
age at serum sampling, median (IQR), y	34.9 (24.2-52)		44.5 (36.8-38.3)	
disease duration at serum sampling, median (IQR), y	6.6 (3.8-12.8)		6.2 (2.8-12.7)	
years of follow-up, median (IQR), y	12.3 (6.4-19)		12.2 (7.6-20.4)	
need of surgery, n (%)	12 (24.0)		4 (8.0)	
treated with biologicals, n (%)	43 (86.0)		16 (32.0)	
**disease location at diagnosis and last follow-up in CD, n (%)**	**diagnosis**	**follow-up**		
L1: ileal	10 (20)	11 (22)		
L2: colonic	10 (20)	13 (26)		
L3: ileocolonic	21 (42)	18 (36)		
L4: upper GI disease	0 (0)	2 (4)		
L1 and L4	0 (0)	1 (2)		
L3 and L4	4 (8)	3 (6)		
unknown	5 (10)	2 (4)		
**disease location in UC, n (%)**			**at diagnosis**	**follow-up**
E1: proctitis			10 (20)	5 (10)
E2: left-sided colitis			18 (36)	21 (42)
E3: extensive (pancolitis)			15 (30)	23 (46)
unknown			7 (14)	1 (2)

CD: Crohn's Disease, UC: Ulcerative colitis, IQR: interquartile range, y: years

Supplementary Table S2. antibody status of CD vs. UC patients

	IBD	CD	UC	p-value*
number of patients	100	50	50	
**ANCA**				
cANCA positivity, n (%)	4 (4.0)	2 (4.0)	2 (4.0)	>0.9
(atypical) pANCA positivity, n (%)	10 (10.0)	6 (12.0)	4 (8.0)	0.741
xANCA positivity, n (%)	31 (31.0)	6 (12.0)	25 (50.0)	<0.001
PR3-ANCA positivity, n (%)	14 (14.0)	2 (4.0)	12 (24.0)	0.008
PR3-ANCA U/ml, median (IQR)	0 (0)	0 (0)	0.8 (0-5.1)	<0.001
MPO-ANCA positivity, n (%)	0 (0)	0 (0)	0 (0)	n.a.
**ASCA**				
IgA positivity, n (%)	40 (40.0)	32 (64.0)	8 (16.0)	<0.001
IgA U/ml, median (IQR)	4 (1.6-15)	9.5 (2.8-83.5)	2.2 (1.5-4.6)	<0.001
IgG positivity, n (%)	23 (23.0)	22 (44.0)	1 (2.0)	<0.001
IgG U/ml, median (IQR)	2 (0.7-7)	5.5 (1.5-43.5)	1 (0.6-2.6)	<0.001
**Antibody Combinations (n (%))**				
xANCA neg, PR3-ANCA neg, all ASCA neg	32 (32.0)	13 (26.0)	19 (38.0)	0.284
xANCA pos, PR3-ANCA pos	9 (9.0)	1 (2.0)	8 (16.0)	0.031
xANCA neg, all ASCA neg	35 (35.0)	13 (26.0)	22 (44.0)	0.093
xANCA neg, any ASCA pos	34 (34.0)	31 (62.0)	3 (6.0)	<0.001
xANCA pos, all ASCA neg	23 (23.0)	3 (6.0)	20 (40.0)	<0.001
xANCA pos, any ASCA pos	8 (8.0)	3 (6.0)	5 (10.0)	0.715
PR3-ANCA neg, all ASCA neg	49 (49.0)	16 (32.0)	33 (66.0)	0.001
PR3-ANCA neg, any ASCA pos	37 (37.0)	32 (64.0)	5 (10.0)	<0.001
PR3-ANCA pos, all ASCA neg	9 (9.0)	0 (0)	9 (18.0)	0.003
PR3-ANCA pos, any ASCA pos	5 (5.0)	2 (4.0)	3 (6.0)	>0.9

*Pearson's Chi-squared test, Fisher's exact test, Wilcoxon rank sum test with correction for multiple testing
IBD, inflammatory bowel disease; CD, Crohn's disease; UC, ulcerative colitis; ANCA, Anti-Neutrophil cytoplasmic antibodies; ASCA, anti-*S. cerevisiae* antibodies; IQR, inter quartile range

Supplementary Figure

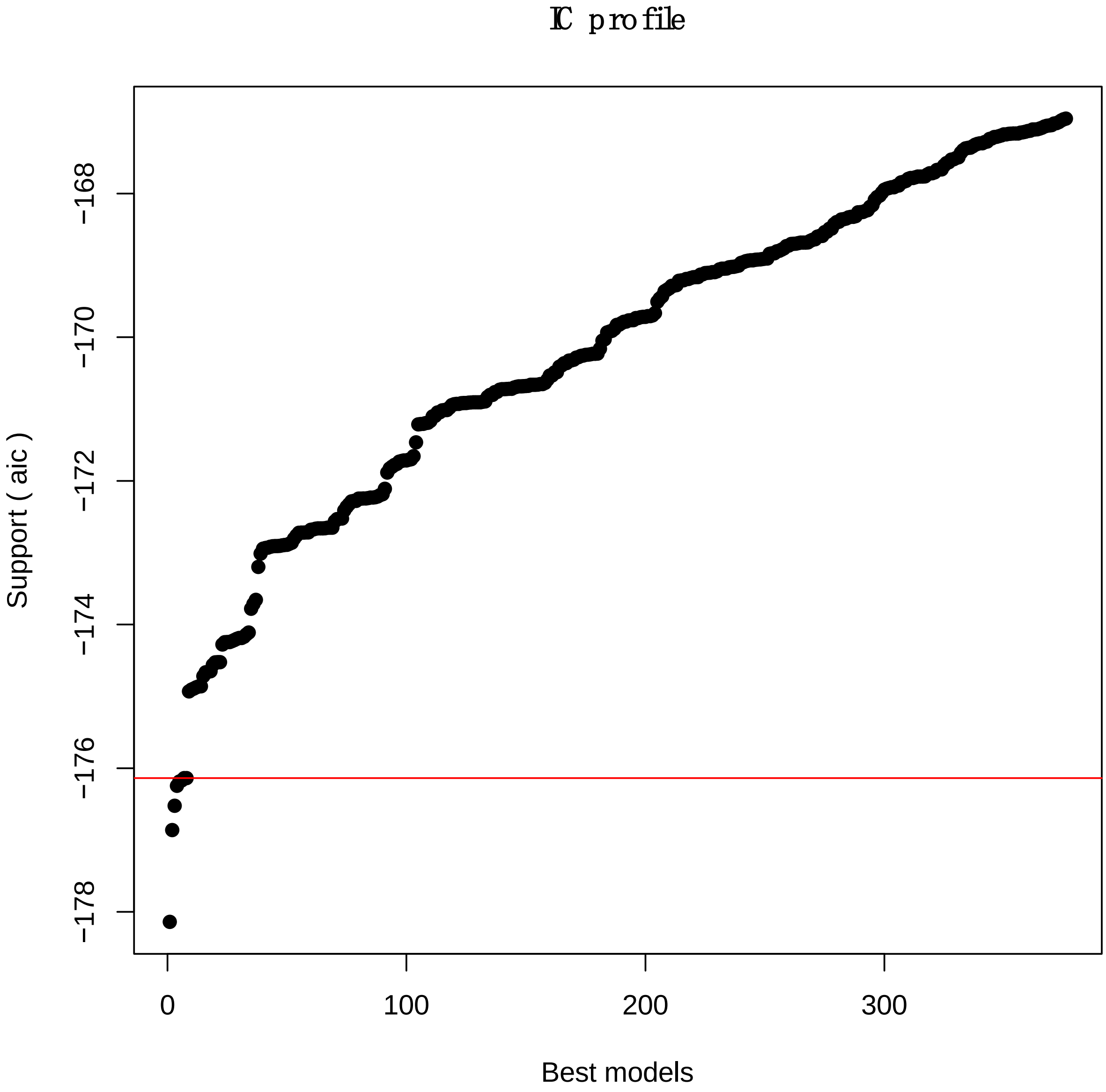

Supplementary Figure S1. Akaike Information Criterion (AIC) profile plot. The horizontal line delineates models that are less than 2 AIC units away from the best model (on the very left). In total, 376 model combinations were assessed.

Figure S2 A,B:

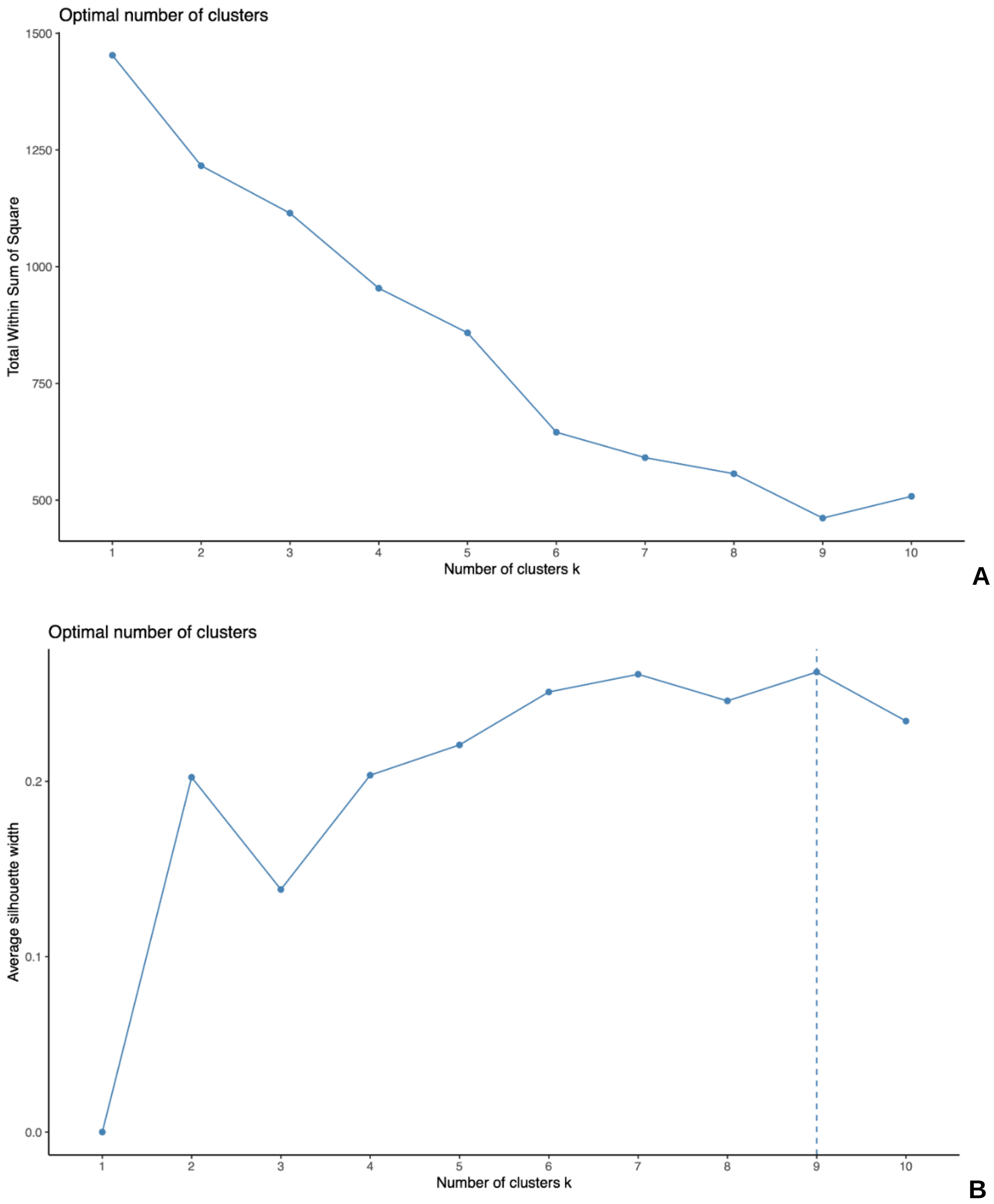

Supplementary Figure S2. Visualization of the number of clusters suggested by the data. Both plots indicate that the true underlying number of clusters is more likely to be two than three. The optimal number of clusters found with this method is nine - though this is clinically not applicable.
